# Supplementary material for: SARS-CoV-2 infection dynamics in Denmark, February through October 2020: Nature of the past epidemic and how it may develop in the future
Source: PLoS One. 2021 Apr 9;16(4):e0249733. doi: 10.1371/journal.pone.0249733 (PMC8034750; doi:10.1371/journal.pone.0249733)
Supplement: S1 File — (DOCX) [file pone.0249733.s001.docx]

**SUPPORTING INFORMATION for:**

**SARS-CoV-2 infection dynamics in Denmark, February through October 2020: Nature of the past epidemic and how it may develop in the future**

Steen Rasmussen^1,2*^, Michael Skytte Petersen^3*^ and Niels Høiby^4,5*^

***Statens Serum Institut data.*** COVID-19 data from Statens Serum Institut [1] is plotted in Fig. 12 (data accessed per August 31, 2020). See caption for details. It should be noted that SSI has modified minor details in data multiple times during the period February through August 2020. However, these changes have not had any measurable impact on our investigations or conclusions.


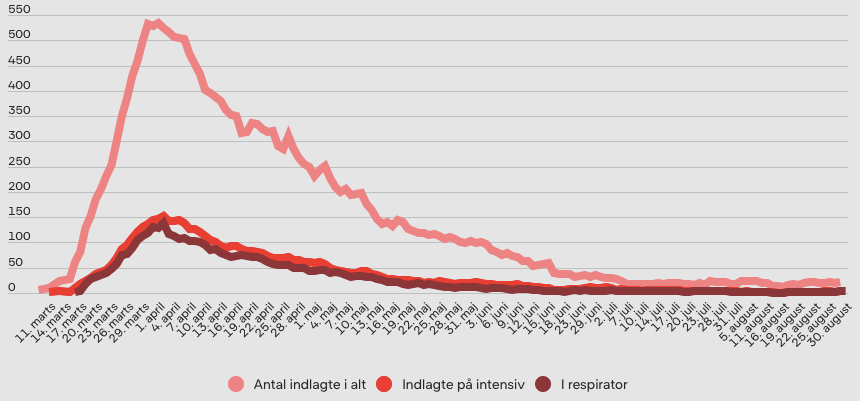

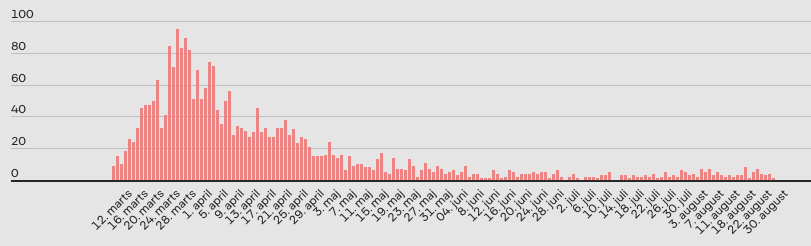


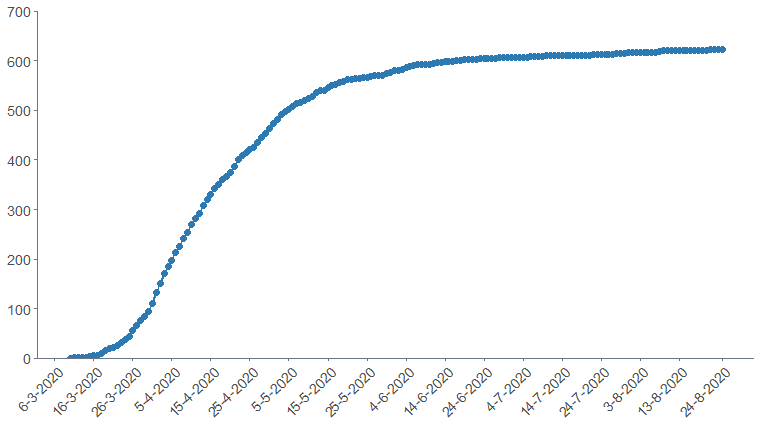

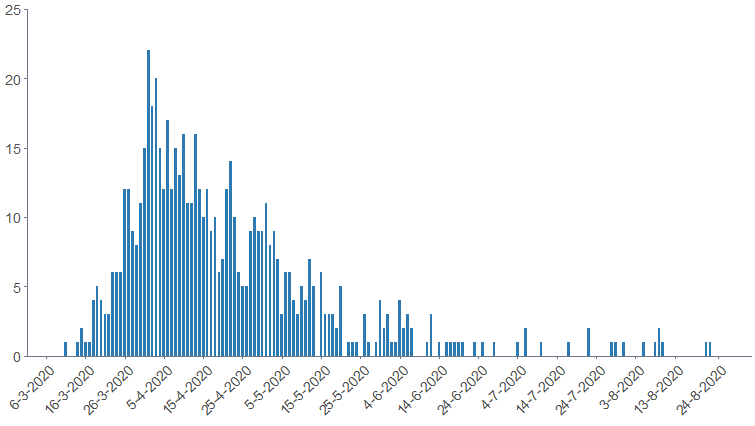


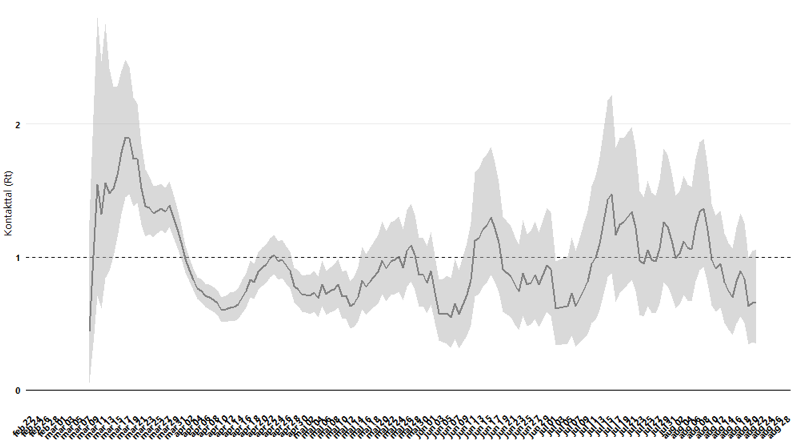

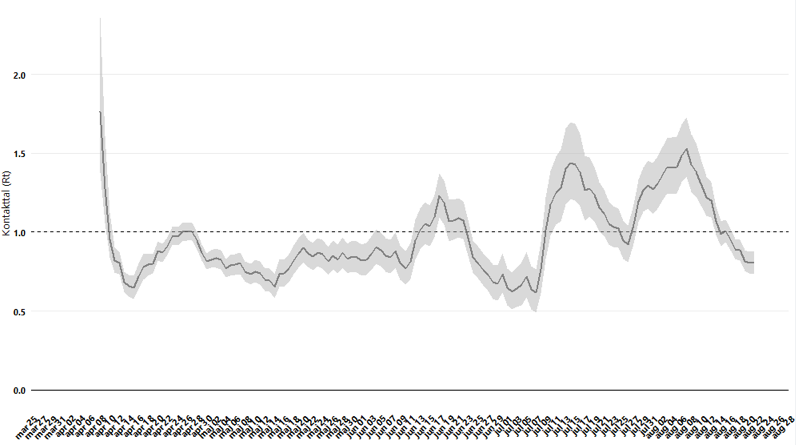


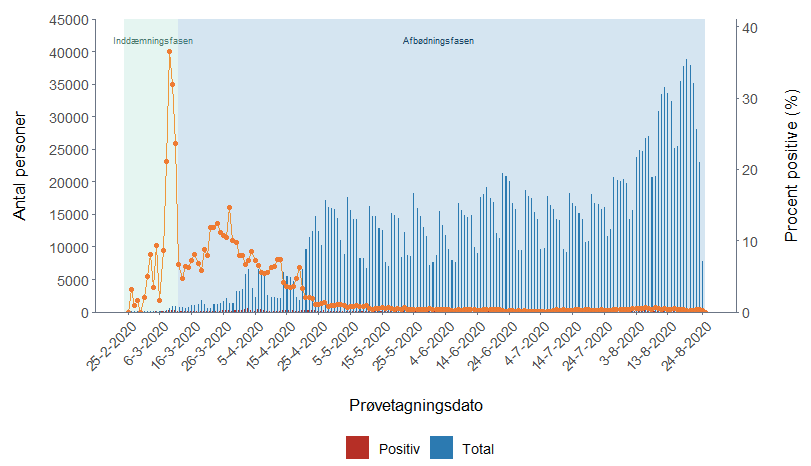

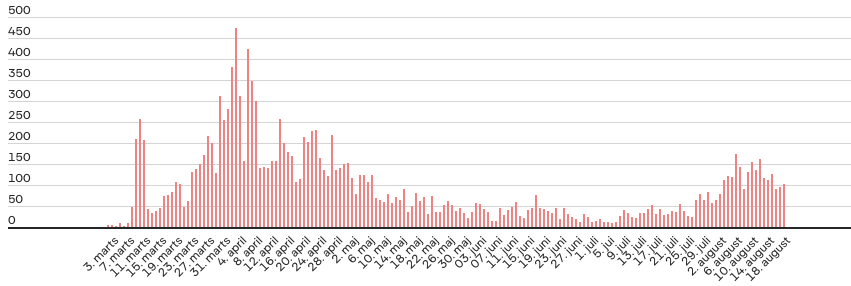


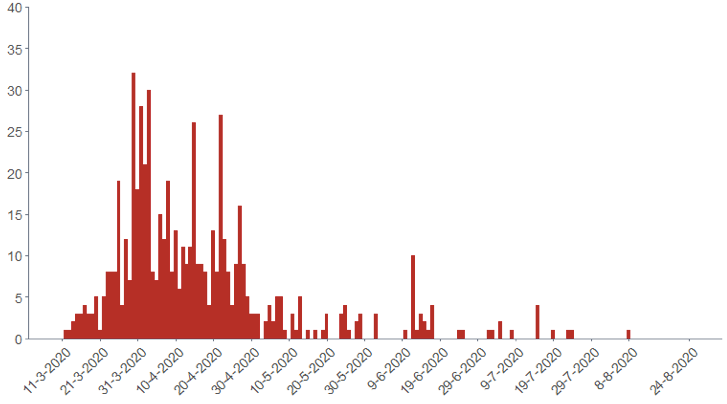


*Figure 12*. *Upper panel*: (1) Daily national COVID-19 hospital occupations and (2) daily admissions. Note the "shoulder" in the admission data around mid-April 2020 indicated by a nearly constant admission level for a couple of weeks that at the time breaks a close to exponential decrease in the pattern of the daily admissions. *Upper middle panel*: (3) accumulated daily deaths and (4) daily deaths. *Lower middle panel*: (5) *ℜ_t_* calculated from the daily hospital admission data and (6) *ℜ_t_* calculated from the nationwide testing program. Note that in the period April 1 - August 31, 2020, one can distinguish five distinct peaks in *ℜ_t_* both measured from the hospital data and from the PCR count. Before about April 1, 2020, the epidemic was dominated by the initial dramatic pandemic growth and successive lockdown of the country making it impossible to identify earlier distinct microscopic events in the data. *Lower, lower middle panel*: (7) daily number of PCR tested nationwide, as well as positively tested and (8) the daily number of positively tested shown at a different scale. Note how the capacity of the national testing program had been stable at around 20,000 tests/day since early May, see image (7), and that the capacity was expanded in August as a result of a local micro-outbreak that started in the city of Aarhus that grew and started to spread to other regions. The national capacity is planned to be expanded to 50-60,000 tests/day by mid fall 2020. *Lowest panel*: (9) the daily number of positive PCR tests at home care centers. Note how increased caution and infection prevention policies over time have significantly decreased the infection level in the older part of the population reflected in the observed number of infected at home care centers.

***Fraction of hospitalized symptomatic infected.*** The current age distribution of the population in Denmark is e.g. given by [2] and by combining it with the age dependent hospitalization fractions given in table 1 yields:

| Age distribution of hospitalization | |
| --- | --- |
| $A0019=1.30/5.83$ | $H_{frac0019}=(0.1+0.3)/2$ |
| $A2039=1.47/5.83$ | $H_{frac2039}=(1.2+3.2)/2$ |
| $A4059=1.56/5.83$ | $H_{frac4059}=(4.9+10.2)/2$ |
| $A6079=1.23/5.83$ | $H_{frac6079}=(16.6+24.3)/2$ |
| $A80up=0.27/5.83$ | $H_{frac80up}=27.3$ |

From these age weighted hospitalization fractions, *h_frac_* is obtained as

$$h_{frac}=(A0019 \times H_{frac0019}+A2039 \times H_{frac2039}$$

$$+ A4059 \times H_{frac4059}+A6079 \times H_{frac6079}$$

$$+ A80up \times H_{frac80up})\frac{1}{100}, Eq. \left( S14 \right)$$

which yields *h_frac_* = 0.0820.

Obviously, the value of *h_frac_* is an important parameter as it scales the epidemic because it determines how many symptomatic infected *I_s_* enter the hospital system, which is the empirical data we use to adjust our simulation. A smaller *h_frac_* implies a larger scale epidemic with higher total number of infected individuals nationwide while a larger *h_frac_* implies a smaller scale epidemic. Note that the overall scale of the epidemic is in part restricted by the empirical hospital and death data and in part by the empirical data from the population wide serological antibody count. A detailed discussion of the impact of the size of *h_frac_* is given below.

***Asymptomatic versus symptomatic SARS-CoV-2 infected persons.*** The percentage of asymptomatic infected persons is according to published data from passengers on ships 45-81% [3][4][5][6] and in children 40% [7]. Further, SARS-CoV-2 positive persons in the last part of their incubation time, before they turn symptomatic thus still being pre-symptomatic, have been shown to harbor the same level of virus RNA in saliva and nasopharyngeal secretions as symptomatic COVID-19 patients and therefore assumed to be nearly as infectious as symptomatic patients [7][8][9][10][11][12][13][14]. This is not the case for incubated persons that later turn asymptomatic.

***Immunological memory*.** During COVID-19 infection an antibody response develops which involves IgM, IgG and IgA. Nearly all patients have developed IgM, IgG and IgA antibodies 3 weeks after onset of the infection [15][16][17][18]. In one study of 70 Chinese patients’ virus neutralizing antibodies correlated with antibodies measured by ELISA test [17], but in a study of 62 European patients only 66% developed protective antibodies as determined by virus neutralisation tests [18]. The duration of the antibody response to SARS-CoV-2 is not known yet, but the experience from the related SARS-CoV infected patients is, that 94% of the patients still had detectable IgG antibodies after 1 year and 50-74% of the patients after 4 years [19][20]. After mild or asymptomatic MERS CoV infection antibodies were either limited or rapidly declined within 3 months whereas the antibody response in severe infections would last for 2-3 years [19][20]. Preliminary results and calculations from China indicate similar dynamics of the SARS-CoV-2 antibody response [21][22][23].

***Infection model***. Note that Eq. (9) can be rewritten as

$$\beta_{i}=\beta_{s}\left( \rho_{s}+\left( 1-\rho_{s} \right)\zeta\delta\right)0.3371, Eq. (S9a)$$

so it has the same form as Eq. (6) with the insertion of an extra factor *δ* = 1.0356. Eq. (S9a) is identical to Eq. (9) for standard parameters, recall Table I. The main difference between this infection model and the simpler infection model given by Eqs. (3) - (6) is a slightly higher level of relative infectiousness for the incubating population for standard parameters. Simulation experiments with both types of infection models convinced us that we could obtain better fits with data if we adopt a higher weight to the incubating population. This is particularly clear when investigating the shape of the initial infection peak in the hospital data.

***Lockdown and re-opening of the country****.* We model the closedown process of the country as a sigmoid function for the infection parameters *β_s_*, *β_a_*, and *β_i_* over a period of approximately 7 days. For all *β_x_*, *x* = *s*, *a*, *i*, as defined in Eqs. (3) - (10) we get

$$\beta_{x}\left( t \right)=\frac{\beta_{s}- d_{x}}{1+e^{-\omega\left( t-t_{0} \right)}}+ d_{x} Eq.(S15)$$

where *ω* defines the steepness of the sigmoid function, *t*_0_ the middle of the approximately 7 days lockdown process, and *d_s_*, *d_a_*, and *d_i_* the infection parameters for *β_s_*, *β_a_*, and *β_i_* immediately after country shut down. Once it was realized that Denmark had been hit by the COVID-19 pandemic, all individuals with flu-like symptoms symptomatic were highly encouraged to self-isolate, so in the following we assume *β_s_* remains at the low level ~ 1% of the free pandemic value, both during lockdown and after the country reopens.

We model the reopening of the country in a manner similar to the closedown process, i.e. by increasing *β_a_* and *β_i_* following the dates where the national reopening policies change. The reopening starts April 20 with the reopening of schools for the youngest kids (K-5) together with a number of small businesses including dentists, hairdressers and other businesses where services are rendered and where close physical proximity between a provider and a costumer is necessary. Later reopening activities were implemented May 7, 20, June 8, and August 14, 2020 [22].

We chose to model the reopening process as a slow (linear) increase of *β_a_* and *β_i_* over that interval. Thus, at time *date_1_*, April 20, 2020, when the country starts reopening activities to *date_2_*, June 8, 2020, a slow linear increase in *β_y_*, *y* = *i*, *a* occurs as defined below:

$$\beta_{y}\left( t \right)=d_{y1}+\frac{d_{y2}- d_{y1}}{{date}_{2}- {date}_{1}}\times\left( t-{date}_{1} \right). Eq. (S16)$$

At time *date_2_*, June 8, 2020, this linear increase levels off and becomes flat with a constant *β_y_^reopen1^* value. For the second reopening, August 14 - 31, 2020, a linear increase is again similarly defined leading to a constant *β_y_^reopen2^* using the August 31, 2020 value.

A graphical depiction of the reopening dynamics as expressed in Eqs. (S15) and (S16) is shown in Fig. 3.

***Estimated parameter values for the simulations.***

We may adjust the *β* parameters in Eqs. (1) and (2) by visual inspection while at the same time ensuring that the scale of the infection fits the data by adjusting *ρ_s_* that determines the relative sizes of the symptomatic and asymptomatic populations. From the national serological test conducted in May 2020, the estimated number of recovered individuals is reported to be about 1.3% of the population (May 28, 2020) although this study only includes individuals of age 18 or above.

Alternatively, we may use a Monte Carlo method (MC) to randomly choose the parameter combinations from a uniform distribution in given intervals in a given scenario. This is done until a desired minimum value of the sum of squared differences (LS) between the daily reported data for total Danish hospital occupations and the same data generated in the corresponding simulation is achieved. Using the MC-LS method we initially need to ensure the optimization process takes into account the appropriate scale of the outbreak that can be obtained by adjusting *ρ_s_*. Thus, we start the formal parameter optimization by initially looping over slightly different *ρ_s_* and different *β_i_^lockdown^* and *β_a_^lockdown^* values followed by a two-step MC-LS process where we first optimize the *β*^0^’s at the onset of the outbreak using the infection model given in Eqs. (7) - (9) followed by an optimization of two parameters simultaneously, *β_a_^lockdown^* and *β_a_^reopen1^* (as well as *β_i_^lockdown^* and *β_i_^reopen1^*).

Going through the simulation with correct seroprevalence numbers for May 28, 2020, the MC solutions with the smallest LS error in the second step is then selected. Then the output from the MC-LS optimization yields the following set of five parameters: *β_s_*^0^, *β_a_^lockdown^*, *β_a_^reopen1^* and thus the associated *β_i_^lockdown^* and *β_i_^reopen1^*. It turns out that the MC-LS method tends to slightly underestimate *β_s_*^0^ compared to a visual inspection mainly because of the historical details of the data. These details data are further discussed in the subsection on “The impact of noise”.

The parameter values from the visual inspection and the MC-LS algorithm are discussed in Figs. 4, 5 and 6, where the main infection parameters are as follows:

Visual inspection: $\beta_{s}^{0}=1.09$, $\beta_{s}^{lockdown}=\beta_{s}^{reopen1}=0.01\times\beta_{s}^{0}$, $\beta_{a}^{lockdown}=0.1019\times\beta_{a}^{0}$ and $\beta_{a}^{reopen1}=0.2305\times\beta_{a}^{0}$.

MC-LS: $\beta_{s}^{0}=1.0583$, $\beta_{s}^{lockdown}=\beta_{s}^{reopen1}=\beta_{s}^{reopen2}=0.01\times\beta_{s}^{0}$, $\rho_{s}=0.1570$, $\beta_{a}^{lockdown}=0.1366\times\beta_{a}^{0}$, $\beta_{a}^{reopen2}=0.2717\times\beta_{a}^{reopen1}$. The MC-LS parameter optimization is done over 10,000 iterations.

The death rates have to be slightly increased to fit the data for the MC-LS simulations (Fig. 6). Other parameter values used are shown in Table I, except the parameters controlling the death rates both from the hospitals and the non-hospital locations.

***Monte Carlo Least Square optimization***

(i) In the first step we optimize the *β*^0^’s at the onset of the outbreak using the infection model given in Eqs. (7) - (9) and optimize for the period February 24 - April 20, 2020. We assume the lockdown *β_s_^lockdown^* 0.01 × *β_s_*^0^ of the outbreak value once the lockdown in a place, as virtually all symptomatic individuals are isolated. We can now optimize for *β_s_*^0^ (as well as *β_a_*^0^ and *β_i_*^0^) in the free outbreak and lockdown period from February 24 to April 20 for different fixed *β_a_^lockdown^* (and *β_i_^lockdown^*) values, where each optimization yields a slightly different *β_s_*^0^: The lower *β_a_^lockdown^* (and *β_i_^lockdown^*), the higher *β_s_*^0^.

(ii) In the second step we optimize two parameters simultaneously, *β_a_^lockdown^* and *β_a_^reopen1^* (as well as *β_i_^lockdown^* and *β_i_^reopen1^*) from April 20 to August 14 with the corresponding *β_s_*^0^ obtained in step (i), also using the infection model given in Eqs. (7) - (9).

The optimization algorithm includes a Monte Carlo method to minimize the sum of squared differences between the Danish hospitalization data and corresponding data generated in simulation, which is the sum of the state variables *H* and *ICU* in the model, recall equations (1) and (2). The Least Squares (LS) difference is defined as follows:

$$Least Squares error=\sum_{i=1}^{n} \left( f\left( t_{i},\Theta\right)-y_{t_{i}} \right)^{2} Eq.(S17)$$

where *y_ti_* = *y_t_*_1_, *y_t_*_2_, ..., *y_tn_* is the observed data and *f* (*t_i_*, Θ) is the corresponding solution of the simulation with a given parameter set Θ = (*θ*_1_, *θ*_2_, ..., *θ_m_*). A pseudo description of the algorithm is as follows:

(i) Generate random numbers within a given domain and choose these numbers as values for selected parameters,

(ii) Run a simulation with these parameter settings,

(iii) Calculate the LS difference between reported and simulated data with chosen parameters,

(iv) If LS error is less than the former LS error then save the parameter values,

(v) Repeat a predefined number of times - or until a certain small LS error value is reached.

*Figure 13* illustrates the Least Squares error for a standard run with ρ_s_ = 0.16 and ζ = 0.309 only sampling on values of β_s_^0^ from a uniform distribution. Note the minimum of the LS error for β_s_^0^ 1.0583.

***The impact of noise***. It may be problematic using our mean field modeling approach to analyze and understand the successive, small and localized epidemic outbreaks over the late spring and summer of 2020. Although these outbreaks are small due to the low national hospital occupation numbers from May through August, most of these later micro-outbreaks are actually reflected in the hospital admittance numbers. Both these earlier (larger) and later (smaller) fluctuations in the hospital data are also picked up in SSI’s daily estimated "contact number" *ℜ_t_* that essentially measures the deviation from the currently observed number of infected compared to an average over that last week [1]. *ℜ_t_* < 1 indicates decreasing observed infected, *ℜ_t_* > 1 indicates increasing observed infected, while *ℜ_t_* ~ 1 means no change in the measured infection level. *ℜ_t_* is estimated both from hospital admittance data and from the observed infected (positively PCR tested) individuals in the population at large. This empirically measured *ℜ_t_* should be distinguished from the theoretically estimated *ℜ_0_*(*t*)*. ℜ_0_*(t) can be derived from our simulation at any *t* and also expresses the average number of infected caused by one infected individual at time *t.*

When minimizing the least square error between the generated total hospital occupation *H* + *ICU* in simulation and the reported total hospital occupation, the fluctuations in the observed data due to localized micro-outbreaks, obscure the ability for the simulated data to match the reported data. This issue can be circumvented by viewing the localized outbreaks as events generated by coincidences that can be implemented by adding a noise term to equation set (1), where the *dS/dt* and *dI_i_/dt* expressions become:

$$\frac{dS}{dt}= -S\left( \beta_{i}I_{i}+\beta_{a}I_{a}+\beta_{s}I_{s} \right)/N$$

$$+ \xi_{s}R_{s} +\xi_{a}R_{a}-Y\left( A\left( t \right),f \right) Eq. (S1a)$$

$$\frac{dI_{i}}{dt}=\frac{S\left( \beta_{i}I_{i}+\beta_{a}I_{a}+\beta_{s}I_{s} \right)}{N}- \gamma_{i}I_{i}+Y\left( A\left( t \right),f \right). Eq.(S1b)$$

Here *Y*(*A*(*t*), *f*) is a Poisson point process with frequency *f* and amplitude *A*(*t*), the $A(t)$ corresponding to a local outbreak of size *A* at time *t*, which on average occurs with frequency *f*. We have included – *Y* (*A*(*t*), *f*) in Eq. (S1a) to maintain population conservation by balancing out the same term in Eq. (S1b). The rest of the Eqs. (1) and (2) remain the same. In the Results Section we review manual parameter adjustment with and without noise as well as the MC-LS parameter optimization without noise.

According to Eqs. (S1a) and (S1b), for April 1 - 20, 2020, we assume *A*(*t*) = 2,500 based on the size of shoulder in the hospital admittance for mid to late April; l (SI Fig. 12 , 5 - 6). *A*(*t*) is thus a bit smaller than 10% of the average size of the total infected population *I*(*t*) = *I_i_* + *I_a_* + *I_s_* in that time interval according to our simulations. For April 21 - August 31, 2020, we assume *A*(*t*) = 1,000 as there are no more major "shoulders" in the data. Thereby *A*(*t*) becomes approximately 1/3 of the size of the total infected population from early July through August, 2020 according to our simulations.

As a basis for the noise added simulation we in part use the standard parameters previously used to generate Figs. 4 and 5: *ρ_s_* = 0.16, *β_s_*^0^ = 1.09 and *β_s_^lockdown^* = *β_s_^reopen1^* = 0.01 × *β_s_*^0^. However, the reopening parameters have to be lower *β_a_^reopen1^* = 0.175 × *β_a_*^0^ and *β_i_^reopen1^* = 0.2305 × *β_i_^0^*. These *β* values are lower so that they, together with the noise added infections, on average generate a similar total number of infections as in the reported data. For simplicity we do not include the last reopening August 14 - 31, 2020, as it does not impact the reported data. Fig. 7 shows a comparison between the observed data and data generated by 100 Monte Carlo simulations each with a different noise sequence realization. Two such noise induced simulations are also highlighted in Fig. 7 to give an impression of the trajectories of individual realizations. Compare Fig. 7 with Figs. 5 and 6.

***Iso-symptomatic infection diagram****.* Quantitatively almost identical iso-symptomatic infection diagrams are found when we Monte Carlo – Least Square optimize the involved parameters not only for the initial free pandemic, but also include data through April 19 (lockdown) or include data through August 14, 2020 (after the first reopening).

In Fig. 8 the "+" curves for *ℜ*_0_ are virtually horizontal, which means that *ζ* and *β_s_*^0^ balance each other out; as *ζ* increases *β_s_*^0^ decreases and vice versa. This can be seen by substituting *ζ* into the expression of *ℜ*_0_ in Eq. (10) that after reducing the expression yields *ℜ*_0_ = 11.5 × *β_s_*^0^ (*ζ*(1 – *ρ_s_*) + *ρ_s_*) where we have also inserted *γ_a_* = *γ_s_* = 0.5 × *γ_i_* = 0.1.

Since all simulations in Fig. 8 approximate the observed initial pandemic we need to empirically identify at least two of the three parameters *ζ*, *β_s_*, *ρ_s_* to know which parameter combination most likely represents the Danish epidemic. We have previously argued for *ζ* = 0.309 in the infection model given by Eqs. (7) - (9).

If we fix *ζ* = 0.309 for the infection model in Eqs. (3) - (6) and use visual inspection we obtain a slightly lower *β_s_*^0^ 1.08 and a higher *ρ_s_* 0.185 to match the initial infection peak in the reported *H_tot_* data as well as the infection scale measured by May 28, 2020 (population wide antibody prevalence). However, with these parameters the later observed data requires a bit higher *β_a_^reopen^*.

If we instead fix *β_s_*^0^ = 1.09 and use visual inspection we obtain *ρ_s_* 0.18 and a slightly higher *ζ* 0.31 that both approximate the observed *H_tot_* data and the observed May 28, 2020 population wide antibody prevalence.

Finally, if we fix *ρ_s_* = 0.16 it becomes more difficult to simultaneously fit the observed *H_tot_* and antibody prevalence as of May 28, 2020. We either obtain a good seroprevalence fit and too low an initial infection wave or a good fit for the infection wave and too high a seroprevalence fit.

The above investigations with different fixed *ζ*, *β_s_*^0^, *ρ_s_* parameters underscore that even though it is always possible to identify a best MC-LS fit to *H_tot_* it might not represent the actual pandemic as it misses the seroprevalence observation - and possibly other observables.

At the onset of the pandemic an often cited *ℜ*_0_ for COVID-19 was ~ 3.2, which is obtained in the diagram if no distinction is made between the symptomatic and asymptomatic infected populations: See *ζ* = 1.0 and *ρ_s_* = 1.0.

Iso-symptomatic infection diagrams could be constructed readily for most infectious diseases. A single diagram would give quantitative information about critical parameters that define the dynamic characteristics of a given epidemic. At a glance, it would also make comparisons between different infectious diseases easier. Similar diagrams where critical thermodynamic parameters of materials are depicted have had tremendous practical importance for the development of engineering since the early days of the industrial revolution.

Figure 8 here.

***Symptomatic versus asymptomatic populations.*** Representative serological tests for SARS-CoV-2 antibodies in a population yields an estimate of the recovered, both symptomatic and asymptomatic, individuals at any given time. These recovery numbers can then be compared to the recovered population generated in simulation. The size of the recovered populations at any given time depends on both the number of recovered infected individuals and on the decay rate of the average immunological memory of symptomatic *ξ_s_* and asymptomatic *ξ_a_* individuals (Eqs. (1)). In principle we need two serological samples at times *t*_1_ and *t*_2_, with some months in between, to fix the parameter values for *ρ_s_* and the average of the two *ξ*’s. Thus, measuring the recovered population at two different times *t*_1_ and *t*_2_ fixes the average *ξ_mean_*, which then can be expressed in days.

Alternatively, we can estimate the immunological memory based on our knowledge of other corona viruses and use these as parameter inputs to our simulations. Assuming a decay time 1/*ξ_s_* to be 700 days and 1/*ξ_a_* to be 60 days respectively, we can determine *ρ_s_* and thus the relative frequency of symptomatic versus asymptomatic individuals from only one randomized serological test at time *t*_1_ [19].

The Danish SARS-CoV-2 seroprevalence was found to be 34 out of 2,424 tested ~ 1.4% of the Danish population during the period May 8 - 28, 2020 [1], where it should be noted that this seroprevalence study only includes adults > 18 years of age. However, about 1/3 of the Danish population had been PCR-tested for SARS-CoV-2 virus by the end of August 2020 and the infection prevalence was found to be ~ 1.1% in adults and ~ 0.6% in children [1]. Since there are ~ 1.30 mil. 0 - 19 aged and ~ 4.52 mil. 20 - 90+ aged in Denmark we estimate 0.6 × (1.30/5.82) × 1.4% + 1.1 × (4.52/5.82) × 1.4% = 1.3692% ~ 1.37%, where we assume the age distribution of infection prevalence is similar to the age distribution in seroprevalence.

We now need to adjust *ρ_s_* to obtain ~ 0.0137 × 5.82 × 10^6^ ~ 79,700 recovered individuals in our simulation by the end of May 2020. This yields a *ρ_s_* 0.16, which means that we estimate that approximately 16% of the infected are symptomatic while 84% of the infected are asymptomatic; thus ~ 5.2 times more asymptomatic than symptomatic infected individuals. Note, as Denmark only has published one prevalence study per August 31, 2020, our estimated scale of the pandemic significantly relies on this one measurement.

***Derivation of ℜ_0_*.** The basic reproduction number, *ℜ*_0_, can be calculated from an epidemic compartment model using the Next- Generation Matrix Method [24]. Our epidemic compartment model given in Eqs. (1) consists of five compartments (*S*, *I_i_*, *I_a_*, *I_s_*, R) with three (*I_i_*, *I_a_*, *I_s_*) infected populations. Letting *S* = *N* (total population) and only including the infected individuals Eqs. (1) reduce to

$$\frac{dI_{i}}{dt}=\beta_{i}I_{i}+\beta_{a}I_{a}+\beta_{s}I_{s}- \gamma_{i}I_{i}$$

$$\frac{dI_{a}}{dt}= \left( 1-\rho_{s} \right)\gamma_{i}I_{i}- \gamma_{a}I_{a}$$

$$\frac{dI_{s}}{dt}= \rho_{s}\gamma_{i}I_{i}-\gamma_{s}I_{s}, Eq. (S18)$$

where we have ignored the hospital dynamics. We may now write Eqs (S18) in matrix form as

$$\frac{dI}{dt}=\left( B+\Gamma\right)I, Eq. (S19)$$

where the *B* matrix express the epidemic transmissions while the Γ matrix express the transitions between the infected populations each defined as

$$B=\left( \begin{matrix} \beta_{i} & \beta_{a} & \beta_{s} \\ 0 & 0 & 0 \\ 0 & 0 & 0 \end{matrix} \right), Eq.(S20)$$

and

$$\Gamma=\left( \begin{matrix} {-\gamma}_{i} & 0 & 0 \\ {\left( 1-\rho_{s} \right)\gamma}_{i} & {-\gamma}_{a} & 0 \\ \rho_{s}\gamma_{i} & 0 & {-\gamma}_{s} \end{matrix} \right). Eq.(S21)$$

*ℜ*_0_ can now be defined as

$$\mathfrak{R}_{0}= \mu\left( B\Gamma^{-1} \right)=\mu\left( K \right) Eq. (S22)$$

where *μ*(*K*) defines the dominating eigenvalue of the resulting *K* matrix.

The inverse of Γ is calculated by $\Gamma^{-1}=\frac{1}{det(\Gamma)}\mathrm{Adj}\left( \Gamma\right).$ The determinant of Γ is

$$\det\left( \Gamma\right)=\gamma_{i}\gamma_{a}\gamma_{s}. Eq.(S23)$$

By first transposing Γ we may calculate the adjunct of Γ by first transposing, then finding the matrix of the minor and then calculating the matrix of cofactors.

$$\Gamma^{T}=\left( \begin{matrix} \gamma_{i} & -{\left( 1-\rho_{s} \right)\gamma}_{i} & {-\rho}_{s}\gamma_{i} \\ 0 & \gamma_{a} & 0 \\ 0 & 0 & \gamma_{s} \end{matrix} \right). Eq.(S24)$$

The matrix of minors is given as

$$M\left( \Gamma^{T} \right)=\left( \begin{matrix} \gamma_{a}\gamma_{s} & 0 & 0 \\ -{\left( 1-\rho_{s} \right)\gamma}_{i}\gamma_{s} & \gamma_{i}\gamma_{s} & 0 \\ \rho_{s}\gamma_{i}\gamma_{a} & 0 & {\gamma_{i}\gamma}_{a} \end{matrix} \right) Eq.(S25)$$

Applying the matrix of cofactors to (37) to obtain the adjunct of Γ

$$\mathrm{Adj}\left( \Gamma\right)=\left( \begin{matrix} \gamma_{a}\gamma_{s} & 0 & 0 \\ {\left( 1-\rho_{s} \right)\gamma}_{i}\gamma_{s} & \gamma_{i}\gamma_{s} & 0 \\ \rho_{s}\gamma_{i}\gamma_{a} & 0 & {\gamma_{i}\gamma}_{a} \end{matrix} \right) Eq.(S26)$$

This implies

$\Gamma^{-1}=\left( \begin{matrix} \frac{1}{\gamma_{i}} & 0 & 0 \\ \frac{\left( 1-\rho_{s} \right)}{\gamma_{a}} & \frac{1}{\gamma_{a}} & 0 \\ \frac{\rho_{s}}{\gamma_{s}} & 0 & \frac{1}{\gamma_{s}} \end{matrix} \right). Eq.(S27)$

By matrix multiplication of *B* and Γ^–1^ we get

$K=B\Gamma^{-1}=\left( \begin{matrix} \beta_{i} & \beta_{a} & \beta_{s} \\ 0 & 0 & 0 \\ 0 & 0 & 0 \end{matrix} \right)\times$ $\left( \begin{matrix} \frac{1}{\gamma_{i}} & -{\left( 1-\rho_{s} \right)\gamma}_{i} & {-\rho}_{s}\gamma_{i} \\ \frac{\left( 1-\rho_{s} \right)}{\gamma_{a}} & \frac{1}{\gamma_{a}} & 0 \\ \frac{\rho_{s}}{\gamma_{s}} & 0 & \frac{1}{\gamma_{s}} \end{matrix} \right)$

= $\left( \begin{matrix} \frac{\beta_{i}}{\gamma_{i}}+\frac{\beta_{a}\left( 1-\rho_{s} \right)}{\gamma_{a}}+\frac{\beta_{s}\rho_{s}}{\gamma_{s}} & \frac{\beta_{a}}{\gamma_{a}} & \frac{\beta_{s}}{\gamma_{s}} \\ 0 & 0 & 0 \\ 0 & 0 & 0 \end{matrix} \right)$

Since *K* = *B*Γ^–1^ is an upper triangular matrix the (only) eigenvalue can be read off of the diagonal and the basic reproduction number is

$$\mathfrak{R}_{0}= \frac{\beta_{i}}{\gamma_{i}}+\frac{\beta_{a}\left( 1-\rho_{s} \right)}{\gamma_{a}}+\frac{\beta_{s}\rho_{s}}{\gamma_{s}} . Eq.(S28)$$

***Estimation of the ℜ_0_ for the different phases of the Danish COVID-19 epidemic*.** *ℜ*_0_ can be estimated for the different phases of the epidemic using Eq. (10) if we adjust the *β_x_*(*t*), *x* = *i*,a*,s* values appropriately. For standard simulation parameters and the simulation started on February 24, 2020 with 690 incubated individuals, we estimate *ℜ*_0_ = 5.361 5.4 for *β_s_*^0^ 1.09 in the free pandemic until the lockdown March 16, 2020, (Figs. 4 and 5). Using a bit lower *β_s_*^0^ 1.06 instead for the MC-LS optimized parameters yields *ℜ*_0_ = 5.213 5.2. During the lockdown and the successive re-opening of the country we assume symptomatic infected individuals are effectively isolated to about 1% of the initial *β_s_*^0^ infection parameter so the majority of the infection transmission occurs due to asymptomatic and incubating individuals. We can derive *ℜ*_0_*^lockdown^* during the lockdown March 16 - April 20, 2020, and *ℜ*_0_*^reopen1^* after the reopening June 8 - August 14, 2020, with infection assumptions either as in Eqs. (3) - (6) or (7) - (9). Using the standard parameters for the simulation (Figs 4 and 5) based on the infection model from Eqs. (7) - (9), we have

$${}_{0}^{lockdown}=\frac{{0.1019\beta}_{i}^{0}}{\gamma_{i}}+\frac{\left( 1-\rho_{s} \right){0.1019\beta}_{a}^{0}}{\gamma_{a}}+\frac{\rho_{s}{0.01\beta}_{s}^{0}}{\gamma_{s}} =0.386 \simeq0.4 \mathrm{and} Eq. (S29)$$

$${}_{0}^{reopen1}=\frac{{0.2305\beta}_{i}^{0}}{\gamma_{i}}+\frac{\left( 1-\rho_{s} \right){0.2305\beta}_{a}^{0}}{\gamma_{a}}+\frac{\rho_{s}{0.01\beta}_{s}^{0}}{\gamma_{s}} =0.8511 \simeq0.9 Eq.(S30)$$

An additional Danish reopening phase occurred over the period August 14 to August 31, 2020, that most significantly allowed physical attendance at higher educational institutions and longer opening times for pubs and restaurants [25]. This last re-opening, however, was partly rolled back in the first half of September due to a significant infection increase. *ℜ*_0_ values from the different phases is illustrated in Fig. 3.

An expanding epidemic requires *ℜ*_0_ > 1. For this to occur after the reopening of the country, the increased infection pressure must almost solely come from the asymptomatic and incubating populations as the symptomatic population is assumed to be almost completely isolated, i.e. *β_s_* = 0.01 × *β_s_*^0^. Taking this value and setting *ℜ*_0_*^reopen1^* = 1.0 in the above equation, we can solve for the factor to multiply *β_a_* and *β_i_* by for the steady state reopening case and find the multiplier to be 0.2717.

From mid-July to mid-August 2020 the noise induced simulations generate a better approximation to the low-level steady state background infection that the observed data indicate. Neither of the deterministic simulations with parameters adjusted either by visual inspection nor by Monte Carlo Least Square optimization are able to capture the balance between the frequent micro-outbreaks and the otherwise slightly decreasing pandemic (Figs. 5, 6, and 7).

An approximate steady state is indicated in the following observables:

(i) a fluctuating low level of hospitalized (*H_tot_* ~ 20) and

(ii) a close to stationary fluctuating number of observed infected (positively PCR tested) individuals in the population at large. The infection numbers fluctuate with an average of 85/day currently based on 20,000 - 40,000 tests/day or 0.3 - 0.6% of the population/day, if we average across the re-occurring micro-outbreaks.

(iii) the two contact numbers *ℜ_t_* fluctuate around 1.0 estimated from the hospital admissions and the national testing program respectively.

We may thus view this situation as a low-level steady state for the background pandemic, with re-occurring, localized, micro-outbreaks, counteracted in part by testing and contact tracing operations. This means that Eq. (S30) should modified to

$${}_{0}^{reopen1}\simeq1.0 Eq. (S31)$$

for July 15 to August 15, 2020 that indicates a quasi steady state epidemic.

***Quasi steady state approximation for populations sizes****.* Recall the hospitalization fraction *h_frac_* 8.2% (SI and (2)), which is the percentage of severely symptomatic infected that need hospital care, recall Eqs. (1) and (2). We can equate *I_s_γ_s_ h_frac_* with the average in-rate to the hospital system consisting of *H_tot_* = *H* + *ICU*. Recall steady state where *dH*/*dt* + *dICU*/*dt* = 0 that can be written out as

$$0={h_{frac}\gamma_{s}I}_{s}-\left( 1-\rho_{h,icu} \right)\gamma_{h}H-\left( 1-\rho_{icu,h} \right)\gamma_{icu}ICU\Leftrightarrow$$

$$I_{s}=\frac{\left( 1-\rho_{h,icu} \right)\gamma_{h}H+\left( 1-\rho_{icu,h} \right)\gamma_{icu}ICU}{h_{frac}\gamma_{s}} Eq.(S32)$$

$$I_{s}=\frac{\left( 1-\rho_{h,icu} \right)\gamma_{h}H_{tot}k+\left( 1-\rho_{icu,h} \right)\gamma_{icu}H_{tot}(1-k)}{h_{frac}\gamma_{s}} ,$$

where in the last equation we have expressed *H* and *ICU* as a function of *H_tot_* where *k* is defined as *H*/*H_tot_*. Inserting the parameter values from Table I we now have an estimate for the symptomatic population *I_s_*. From this we can also estimate the other infected populations as:

$$I_{a}=\left( \frac{\left( 1-\rho_{s} \right)}{\rho_{s}} \right)I_{s} \mathrm{and} Eq. (S33)$$

$$I_{i}=\frac{\left( I_{a}+I_{s} \right)}{2} Eq.(S34)$$

where the last relationship follows from *γ_a_* = *γ_s_* = 0.5*γ_i_*. Thus, the size of the total infected population *I_tot_* is:

$$I_{tot}=I_{i}+I_{a}+I_{s}. Eq.(S35)$$

Currently PCR tests should be able to pick up SARSCoV-2 RNA starting the last 1.5 days of the ~ 5 day incubation period and through the ~ 10 day period where the infected individuals are either symptomatic or asymptomatic.

Still assuming *γ_a_* = *γ_s_* = 0.1/day and no PCR detection after the 10-day infection period associated with *I_s_* and *I_a_*, the detectable steady state population is approximately

$$I_{tot}={0.3 \times I}_{i}+I_{a}+I_{s ,} Eq. (S36)$$

as *I_i_* on average can only be identified the last 1.5 days of the 5 day incubation time; thus the factor 0.3. It should be noted, however, that RNA virus can be excreted and be picked up by PCR measurements significantly longer than the approximately 10 days we have assumed as the duration of the infection both for the symptomatic and asymptomatic cases [23].

From Eqs. (1) for the steady state case we note that

$$\frac{dI_{i}}{dt}=\frac{\left( \beta_{i}I_{i}+\beta_{a}I_{a}+\beta_{s}I_{s} \right)S}{N}- \gamma_{i}I_{i}=0. Eq.(S37)$$

In steady state this means that the two terms should be identical; what goes into *I_i_* is equal to what goes out of *I_i_*. Thus, we can estimate the daily rate at which newly infected are recruited from the susceptible population as

$$I_{new}=\gamma_{i}I_{i .} Eq. (S38)$$

Based on hospitalization data and using Eqs. (S32) to (S38), we can estimate the corresponding infected and observable populations sizes as well as the daily infection rate, none of which are directly observable.

Note the linear proportionality between the numbers; e.g. 10 times higher hospital population yields 10 times higher infected populations and infection rates. Also note that these estimates may be impacted by testing and removal of identified infected individuals so their ability to further spread the infection is reduced. Finally, it should be noted that quasi steady state approximations are often successfully used even when true steady state conditions do not yet apply.

During the quasi steady state period from mid July to mid August, 2020, Denmark on average had 20 hospitalized COVID-19 patients (*H_tot_*) where the intensive care unit patients are approximately one fifth of every patient, *ICU* = 0.2 × *H_tot_* = (1 – *k*) × *H_tot_*. Inserting these numbers and default values from Table I into Eqs. (S32) to (S36) and (S38) we obtain the following population infection numbers:

$$I_{s}\simeq\frac{\left( 1-0.135 \right)\times20\times\frac{1}{8}\times0.8+\left( 1-0.72 \right)\times20\times\frac{1}{10}\times0.2}{\frac{1}{10}\times0.082}$$

225

*I_a_* (0.84/0.16) × 225 1179

*I_i_* (225 + 1179)/2 702

*I_tot_* 225 + 1179 + 702 2106 Eq. (39)

*I_obs_* 225 + 1179 + 0.3 × 702 1615

*I_new_* 702 × 0.2 140

The reopening of the country August 14 - 31, 2020 unfortunately caused a new significant infection rise with an increase in the hospitalizations from early to late September. The infection rise initially had a doubling time of approximately 8 days and by the end of September, 2020 the hospital occupation largely leveled off around 110 due to a number of national initiatives including renewed information campaigns and reinstated restrictions including early closing of bars (10 pm) and at most 50 people at private events. If we assume a quasi steady state for October 1 - 20, 2020 we get from (11) the averages *H_tot_* = 111, *k* = 0.85 and *T_d_* = 407 that inserted in the Eqs. (S32) – (S38) yield:

*I_i_*  4,606

*I_a_* 6,830

*I_s_* 1,301 Eq.(S40)

*I_tot_* 12,196

*I_obs_* 9,351 and

*I_new_* 813

The key population numbers for different *H_tot_* population sizes are tabulated in Table II (all numbers are calculated on a computer with double precision and then rounded to integer values).

***Influenza infection pattern***


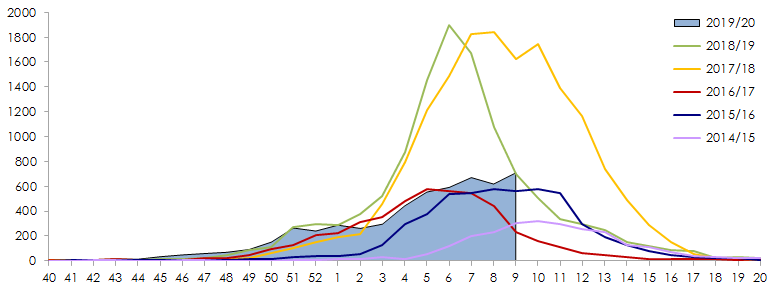


*Figure 14*. Vertical axis: number of laboratory verified influenza cases in Denmark shown for the flu seasons 2014/15 to 2019/20 until the onset of the COVID-19 pandemic. Horizontal axis: week number in a calendar year. Note the clear yearly epidemic pattern that starts in the early winter and peaks between early February and mid-March. It should also be noted that the closing of Denmark due to COVID-19 March 16, 2020, stopped the influenza epidemic and actually also a smaller whooping cough epidemic, not shown in the above graph [26].

***Impact of the fraction of hospitalized symptomatic population****.* Because *h_frac_* is a critical part of our population estimates, we investigate in simulation the impact of both a lower *h_frac_* = 0.061 and a higher *h_frac_* = 0.139 using visual inspection for appropriate fits between reported *H* and *ICU* occupation data as well as death data and the corresponding values generated in simulation. Recall the original *h_frac_* = 0.082.

A modified value for *h_frac_* requires a significant re-adjustment of the main infection parameters. If *h_frac_* increases, a larger fraction of the symptomatic infected *I_s_* needs hospitalization. As the reported hospitalization volume at any given time does not change, this implies that the total number of symptomatic infected *I_s_* must also be smaller to ensure correspondence between the reported and simulated data. Thus, increasing *h_frac_* means a decreasing *ρ_s_* so a smaller fraction of incubated *I_i_* becomes symptomatic *I_s_* while a larger fraction (1 – *ρ_s_*) becomes asymptomatic *I_a_*. As we know from the COVID-19 iso-symptomatic infection diagram investigation, a smaller *ρ_s_* means a larger *β_s_*^0^ and a larger *ℜ*_0_ for a constant *ζ* value, that in our case is set to 0.309 As *β_s_*^0^ and *ℜ*_0_ change for the initial free pandemic so must *β_x_^lockdown^*, *β_x_^reopen1^*, *β_x_^reopen2^*, *x* = *i*, *a* and their corresponding *ℜ*_0_ values.

Conversely, a smaller *h_frac_* implies a larger *ρ_s_* and a smaller *m_frac_* as well as smaller *β_s_* and *ℜ*_0_.

Hospital occupation and death data fits can be obtained both for *h_frac_* = 0.061 and 0.139, but only for the *h_frac_* = 0.139 case is it in the same simulation also possible to obtain the correct seroprevalence number of 79,700 or 1.37% of the adult population per May 28, 2020. For *h_frac_* = 0.061 the simulated seroprevalence is 96,700 and thus overshoots with a factor of 96,800/79,700 1.21, if we at the same time require appropriate fits for *H*, *ICU* and deaths tolls.

In more details: If *h_frac_* increases a larger fraction of the symptomatic infected *I_s_* needs hospitalization. As the reported hospitalization volume at any given time does not change, this implies that the total number of symptomatic infected *I_s_* must also be smaller so as to ensure correspondence between the reported and simulated data. Thus, increasing *h_frac_* means a decreasing *ρ_s_* so a smaller fraction of incubated *I_i_* becomes symptomatic *I_s_* while a larger fraction (1 – *ρ_s_*) becomes asymptomatic *I_a_*. As we know from the COVID-19 iso-symptomatic infection diagram investigation, a smaller *ρ_s_* means a larger *β_s_*^0^ and a larger *ℜ*_0_ for a constant *ζ* value, that in our case is set to 0.309. As *β_s_*^0^ and *ℜ*_0_ change for the initial free pandemic so must *β_x_^lockdown^*, *β_x_^reopen1^*, *β_x_^reopen2^*, *x* = *i*, *a*, and their corresponding *ℜ*_0_ values.

We test two concrete *h_frac_* values away from the standard *h_frac_* = 8.2%:

(i) *h_frac_* = 13.9%, a ~ 70% increase, see Fig. 15.

(ii) *h_frac_* = 6.1%, a ~ 25% decrease, see Fig. 16. Figs. 15 and 16 should be compared with Figs. 4, 5 and 6.

Although both simulation experiments in Figs. 15 and 16 generate nice correspondence with the observed primary data, only *h_frac_* = 13.9% is simultaneously able to satisfy the reported seroprevalence of May 28, 2020. This means that 0.082 ≤ *h_frac_* ≤ 0.139 can satisfy all observed data with parameter adjustments, while *h_frac_* = 6.1% cannot. See more details in the figure captions.

*Fig 15.* *Upper panel*: Reported and simulated hospital *H_tot_*, *ICU* and death data for *h_frac_* = 13.9% linear scale. Simulated seroprevalence matches the observed May 28, 2020 value. Necessary parameter adjustments compared to standard simulation, recall Table I. Epidemic parameters: *β_s_*^0^ = 1.244, *ρ_s_* = 0.09, *β_a_^lockdown^* = 0.09 × *β_a_*^0^, *β_a_^reopen1^* = 0.193 × *β_a_*^0^, *β_a_^reopen2^* = 0.228 × *β_a_*^0^. As expected, the fraction of terminally ill non-hospital patients is significantly increased *m_frac_* = 0.0131, while the hospital system parameters are only slightly changed: *ρ_h,r_* = 0.78, *ρ_h,icu_* = 0.135, *ρ_h,d_* = 0.085. The rest of the simulation parameters are the standard values given in Table I. *Lower panel*: Same reported and simulated observables but logarithmic scale. Dots represent the observed hospital and death data, lines represent the simulation data.

*Fig 16.* *Upper panel*: Reported and simulated hospital *H_tot_*, *ICU* and death data for *h_frac_* = 6.1% linear scale. Simulated seroprevalence of ~ 96,400 exceeds observation of ~ 79,700 May 28, 2020. Necessary parameter adjustments compared to standard simulation, recall Table I. Epidemic parameters: *β_s_*^0^ = 1.085, *ρ_s_* = 0.183, *β_a_^lockdown^* = 0.11 × *β_a_*^0^, *β_a_^reopen1^* = 0.225 × *β_a_*^0^, *β_a_^reopen2^* = 0.277 × *β_a_*^0^. No adjustments of the health care parameters are necessary, but as expected *m_frac_* = 0.0058 is significantly lower because *I_s_* becomes larger due to the smaller *h_frac_*. *Lower panel*: Same reported and simulated observables but logarithmic scale. Dots represent the observed hospital and death data, lines represent the simulation data.

**References**

1. <https://www.ssi.dk>. Outbreak of COVID-19. Numbers and Surveillance of COVID-19. 2020.
2. <https://www.statista.com/statistics/570654/total-population-in-denmark-by-age/>. Total population in Denmark by age 2020.
3. Russell T, Hellewell J, Jarvis C, Zandvoort K, Abbott S, Ratnayake R, et al. Estimating the infection and case fatality ratio for COVID-19 using age-adjusted data from the outbreak on the Diamond Princess cruise ship. medRxiv preprint 2020.
4. Wikipedia. COVID-19 pandemic on Diamond Princess. https://en.wikipedia.org/w/index.php?title=COVID-19 Wikipedia; 2020
5. Ing AJ, Cocks C, Green JP. COVID-19: in the footsteps of Ernest Shackleton. Thorax 2020;75:693-694.
6. Kasper M, Geibe J, Sears C, Riegodedios A, Luse T, von Thun A, et al. An outbreak of Covid-19 on an aircraft carrier. NEJM 2020.
7. Zheng B, Wang H, Yu C. An increasing Public Health burden arising from children infected with SARS-CoV-2: a systematic review and metaanalysis. Pediatr Pulmonol 2020, Aug. 5 (doi: 10.1002/ppul. 25008)
8. Amendola A, Tanzi E, Folgori L, Barcelline L, Bianchi S, Gori M, et al. Low seroprevalence of SARS-CoV-2 infection among healthcare workers of the largest children hospital in Milan during the pandemic wave. Doi 10.1017/ice.2020.401. Infect Control Hospital Epidemiol. 2020:1-2.

9. Dora A, Winnett A, Jatt L, Davar K, Watanabe M, Sohn, et al. Universal and serial laboratory testing for SARS-CoV-2 at a long-term care skilled nursing facility for veterans Los Angeles, California, 2020. Morbidity and Mortality Weekly Report. 2020; 69 (May 29):651-5.

10. Wang Y, Tong J, Qin Y, Xie T, Li J, Li J, et al. Characterization of an asymptomatic cohort of SARS-CoV-2 infected individuals outside Wuhan, China. Clin Infect Dis. 2020.

11. Li G, Li W, He X, Cao Y. Asymptomatic and presymptomatic infectors: Hidden sources of COVID-19 disease. Clin Infect Dis 2020.

1. Baggio S, L’Huillier A, Yerly S, Bellon M, Wagner N, Rohr M, et al. SARS-CoV-2 viral load in the upper respiratory tract of children and adults with early acute COVID-19. Clinical Infectious Diseases 2020
2. Campioli C, Cevellos E, Assi M, Patel R, Binnicker M, O’Horo J. Clinical predictors and timing of cessation of viral RNAQ shedding in patients with COVID-19. J Virol 2020.
3. Chau N, Lam V, Dung N, Yen L, Minh N, Hung L, et al. The natural history and transmission potential of asymptomatic SARS-CoV-2 infection. Clin Infect Dis. 2020.
4. Wu J-L, Tseng W-P, Lin C-H, Lee T-F, Chung M-Y, Hunag C-H, et al. Four point-of-care lateral flow immunoassays for diagnosis of COVID-19 and for assessing dynamics of antibody responses to SARS-CoV-2. J Infect 2020.

16. Yu H-Q, Sun B-Q, Fang Z-F, Zhao J-C, Liu X-Y, Li Y-Mea. Distinct features of SARS-CoV-2-specific IgA response in COVID-19 patients. Eur Respir J 2020.

17. Chew K, Tan S, Saw S, Pajarillaga A, Zaine S, Khoo C, et al. IgG antibody response on the Abbott Architect for established SARS-CoV-2 infection. Clin Microbiol Infect 2020.

18. Jääskeläinen A, Kuivanen E, Kekäläinen E, Ahava M, Loginov R, Kailio-Kokko H, et al. Performance of six SARS-CoV-2 immunoassays in comparison with microneuralization. . J Clin Virol 2020;129:1-10.

19. Yin S, Tong X, Huang A, Shen H, Li Y, Liu Y, et al. Longitudinal anti-SARS-CoV-2 antibody profile and neutralization activity of a COVID-19 patient. J Infect. 2020;81(3).

20. Lin Q, Zhu L, Ni Z, Meng H, You L. Duration of serum neutralizing antibodies for SARS-CoV-2: lessons from SARSCoV infection. J Microbiol Immunol Infect 2020.

21. Kellan P, Barclay W. The dynamics of humoral immune responses following SARS-CoV-2 infection and the potential for reinfection. J Gen Virol. 2020.

22. Wang K, Long Q-X, Deng H-J, Hu J, Gao Q-J, Zhang G-J, et al. Longitudinal dynamics of the neutralising antibody re-sponse to SARS-CoV-2 infection. Clin Infect Dis. 2020.

23. He Z, Ren L, Yang J, Guo L, Feng L, Ma C et al. Seroprevalence and humopral immune response durability of anti-SARS-CoV-2 antibodies in Wuhan, China: a longitudinal, populations-level, cross-sectional study. Lancet 397:1075-84; 2021.

24. Diekmann O., Heesterbeek J.A.P., Roberts M.G., The construction of next-generation matrices for compartmental epidemic models, *J.R. Soc. Interface* 2010, Vol. 7, p873-885, doi:10.1098/rif.2009.0386.

25. <https://politi.dk/coronavirus-i-danmark/kontrolleretgenaabning-af-danmark>. Coronavirus i Danmark 2020.

26. Statens Serum Institut. Influenzasæsonen 2019/2020. EPI-NYT. 2020; uge 26 (week 26).
