# Supplementary material for: SARS-CoV-2 infection dynamics in Denmark, February through October 2020: Nature of the past epidemic and how it may develop in the future
Source: PLoS One. 2021 Apr 9;16(4):e0249733. doi: 10.1371/journal.pone.0249733 (PMC8034750; doi:10.1371/journal.pone.0249733)
Supplement: Attachment — Submitted filename: Response to Reviewers.pdf [file pone.0249733.s002.pdf]

Response to Reviewers March 5, 2021

Dear Editors for PLOS ONE,

Please see below how we address all reviewer comments:

Reviewer 1 states:

"… The study itself uses a highly specialized mathematical language that makes it difficult to adjust to the language normally used by non-mathematic readers. The different parts of the manuscript are too long: introduction, methods, results and conclusions. It is very difficult to read (again, for non-mathematics and related areas involved). Maybe data could be used in other scenarios with different population rates, but I'm not sure if could be used in others health care systems. I consider it is an excellent manuscript, but most readers will not be mathematicians, so reading can be very difficult…"

The main critique is along two different lines: (i) The text is too mathematical and (ii) the paper is too long.

To address these two issues, we have: (a) removed most of the mathematical details and arguments from the main text (Manus) and added these more technical text section to the Supplementary Information document. (b) We have also rewritten multiple parts of the main text (Manus) to make our analysis is more intuitive and less technical. Alle changes are indicated with red font.

The upside of these many changes is two-fold: (c) The manus text is significantly easier to read for medical scientists and (d) the manus text is much shorter. The "Methods" section is reduced by 39% and the "Results" section is reduced by 41%.

We sincerely hope these improvements will satisfy the main critique from the Reviewer 1.

Reviewer 1 further questions whether our developed methods and models can be used for health care systems in different regions and countries.

Reviewer 2, however, states: *"… this is a well designed and written study which can provide useful insights about the nature of the pandemic in other countries and regions …"*

Thus, the two reviewers do not agree on this issue.

In the subsection entitled "Hospitalization and estimated populations in other regions" we re-emphasize that further verification would be needed to know exactly how well our approach work in any particular region. However, most countries have a hospital system similar to the Danish with regular hospital sections and intensive care units (ICU) where only the very ill move to the ICU. Therefore, we still believe our approach could also provide useful insight about the pandemic in other regions

Sincerely yours,

Steen Rasmussen
Center for Fundamental Living Technology (FLinT)
Department for Physics, Chemistry and Pharmacy, University of Southern Denmark

Santa Fe Institute, Santa Fe, NM 87501, USA
steen@sdu.dk

Michael Skytte Petersen
Center for Biosecurity and Biopreparedness (CBB), Statens Serum Institut, Denmark
mspe@ssi.dk

Niels Høiby
Department of Clinical Microbiology, Rigshospitalet, Copenhagen, Denmark
Institute of Immunology & Microbiology, Panum Institute, University of Copenhagen, Denmark
hoiby@dadlnet.dk
